# Practical Urinalysis

**Published:** 1898-12

**Authors:** 


PRACTICAL URANALYSIS AND URINARY DIAGNOSIS: A Manual for the use of Physicians, Surgeons, and Students. By Charles W. Purdy, M. D., LL. D. (Queen's University); Fellow of the Royal College of Physicians and Surgeons, Kingston; Professor of Clinical Medicine at the Chicago Post-Graduate Medical School. Author of "Bright's Disease and Allied Affections of the Kidneys;" also of "Diabetes: Its causes, Symptoms, and Treatment." Fourth revised edition. With numerous illustrations, including photo-engravings and colored plates. In one crown octavo volume, 365 pages, bound in extra cloth, $2.50 net. The F. A. Davis Co., Publishers, 1914-16 Cherry Street, Philadelphia; 117 West Forty-second Street, New York City; 9 Lakeside Building, 218-220 South Clark Street, Chicago, Ill.

This is the fourth edition of this valuable book. It has been reviewed in these pages in every edition that has been published, and we have had only unqualified commendation to pass upon it every time we have noticed it.

An elaborate review of the work was published in this journal for August, 1895, page 384. In that review were given the most improved methods of examining urine for albumen and sugar as perfected by the author. To that review the reader is referred for more definite information concerning the work.

Purdy's book has been adopted as a text book in more than sixty medical colleges, and has now passed into the fourth edition, as before stated.

Considerable change has been made in the chemical part to bring the work up to the present time. Obsolete methods have been omitted in order to make room for these latest and most improved tests. There has been a number of illustrations added that still further increase the value of the book.

We give this work the same unqualified endorsement that we have in the past to the other editions.

